# Borders of physical self in virtual reality: a systematic review of virtual hand position discrepancy detection

**DOI:** 10.3389/fpsyt.2024.1455495

**Published:** 2025-01-06

**Authors:** David Antoš, Tomáš Švec, Jana Hořínková, Eliška Bartečková

**Affiliations:** ^1^ Department of Psychiatry, Faculty of Medicine, Masaryk University, Brno, Czechia; ^2^ Department of Computer Graphics and Multimedia, Faculty of Information Technologies, Brno University of Technology, Brno, Czechia

**Keywords:** virtual reality, body ownership, hand redirection, bodily self-consciousness, self-location, just noticable difference, point of subjective equality, detection threshold

## Abstract

**Introduction:**

Virtual reality (VR) holds significant promise for psychiatric research, treatment, and assessment. Its unique ability to elicit immersion and presence is important for effective interventions. Immersion and presence are influenced by matching—the alignment between provided sensory information and user feedback, and self-presentation—the depiction of a user’s virtual body or limbs. Discrepancies between real and virtual hands can affect the sense of presence and thus treatment efficacy. However, the precise impact of positional offsets in healthy individuals remains under-explored. This review assesses how various factors influence the detection thresholds for positional offsets in VR among healthy subjects.

**Methods:**

A comprehensive database search targeted English-language studies on the detection thresholds of virtual hand positional offsets using head-mounted displays (HMDs) with specific tracking capabilities. Data on methodologies, participant demographics, and VR system specifics were extracted.

**Results:**

Thirteen studies met the inclusion criteria, revealing significant variability in detection thresholds—from a few millimeters to 42 cm for linear shifts and from 2° to 45° for angular shifts. Sensitivity to these offsets was affected by hand movement direction and magnitude, hand representation realism, and the presence of distractions. VR system specifications, such as resolution and tracking accuracy, also played a significant role. Methodological issues included small sample sizes, inadequate demographic reporting, and inconsistent presence or avatar embodiment measures.

**Conclusion:**

The results highlight the need to consider identified influencing factors to maximize user presence in VR-based therapies. Variability in VR device capabilities also emphasizes the need for detailed reporting of device properties in research. The individual variability in offset detection further illustrates VR’s potential as a tool for studying body ownership and multisensory integration.

## 1 Introduction

Virtual reality (VR) is a technology that has been attracting the attention of researchers in psychiatry and neuroscience since the 1990s (1), soon after Jaron Lanier first used the term in 1987 (2). Interest in this technology was increasing hand in hand with advances in computer graphics capabilities and, in recent years, also with increased availability of cost-effective VR hardware. The main feature distinguishing this human-computer interaction method from others is the ability to elicit a sense of presence in the user (3). However, this is only one of its advantages, which further include options to present various multi-modal stimuli, manipulate them and measure the activity of the user in a simulated environment (4). VR thus represents an unparalleled tool for ecologically valid treatment interventions and assessment.

Presence is mediated by but distinct from the concept of immersion. While presence is a subjective feeling of ‘being there’ within the virtual environment, immersion is an objective measure of the hardware’s capabilities (5). The degree of immersion—and consequently, the sense of presence—is influenced by several facets of VR equipment. These aspects include *matching*, which refers to the concordance between provided sensory information and the user’s feedback, and *self-presentation*, involving the representation of the participant’s virtual body or limbs (5). The main focus of this review will be on matching, with self-presentation discussed primarily as an influencing factor.

To achieve a satisfactory sense of presence, the position of the user’s virtual self-representation needs to align closely with that of their real body or limbs. However, phenomena such as the rubber hand illusion (6), indicate that this alignment does not need to be perfect. There appears to exist a perceptible margin within which discrepancies between the virtual and actual limb positions do not significantly disrupt the user’s sense of immersion and presence. For the purpose of this review, the discrepancy between the virtual and actual limb positions will be termed *positional offset*.

The ability to detect and respond to positional offsets of a virtual hand can significantly affect a user’s sense of immersion and presence. In the following text, the term *detection threshold* will refer to the minimum spatial difference between the virtual and real hands that can be detected with a predefined probability. Understanding detection thresholds can help optimize VR environments to enhance immersion, making therapeutic interventions more effective. Additionally, detection thresholds can serve as a practical, technology-agnostic proxy measure to address standardization of various VR hardware in mental-health research. Lastly, variability in positional offset detection offers a promising research tool for exploring alterations in bodily self-consciousness and related constructs.

Bodily self-consciousness represents the integration of various sensory inputs to construct the physical self (7). It outlines three main components: sense of ownership, self-location, and first-person perspective (8). Sense of ownership refers to the feeling that a body or body part belongs to oneself (9) and self-location is the perceived spatial location of oneself (10). The first-person perspective is the viewpoint from which one interacts with the world (11), although some argue it cannot be distinctly separated from self-location (8).

Disturbances in body ownership and self-location are prevalent in several mental disorders, indicating altered bodily self-consciousness. Notably, this includes schizophrenia spectrum disorders (12), borderline personality disorder (13), eating disorders (14), social anxiety (15) post-traumatic stress disorder (16) and autism spectrum disorders (17). Exploring interindividual variability in positional offset detection could illuminate underlying mechanisms of bodily self-consciousness alterations and contribute to a development of diagnostic tools and targeted interventions.

While basic concepts such as immersion and presence in VR are well-established, the precise impact of positional offsets on these experiences in healthy individuals has not yet been thoroughly quantified. Understanding the detection thresholds and influences of positional offsets is essential for creating immersive VR environments and for body-consciousness research. This review seeks to fill this gap by systematically assessing how various factors affect the perception of positional shifts, thereby providing a reliable baseline from which deviations in clinical populations can be identified and addressed.

The aim of this review therefore is to assess the detection thresholds for positional offsets in virtual reality among healthy individuals, examining how factors such as the direction of movement, temporal characteristics, visual representation, and environmental distractions influence these thresholds.

## 2 Methods

### 2.1 PRISMA checklist

This systematic review was conducted with regard to the Preferred Reporting Items for Systematic reviews and Meta-Analyses (PRISMA) (18). The PRISMA 2020 checklist, along with item locations, is provided in the **Supplementary Materials**.

### 2.2 Eligibility criteria

This systematic review included research articles, proceeding papers, and book chapters published in English from 1998 to 2024. The starting year marks the first mention of the Virtual Research V8 VR set in a published study. Only completed studies that presented original data were considered. Exclusions were made for pilot studies, reviews, abstracts, and studies involving neurologically or psychiatrically impaired participants.

For inclusion, studies had to employ immersive virtual reality technology, characterized by the immersion of participants in a 3D virtual environment equipped with a head-mounted display and a hand-tracking system. Technologies involving mixed reality, shutter glasses, and virtual scenarios displayed on a computer screen were excluded.

Eligible studies had to involve a positional shift applied to a virtual hand. These shifts could be angular or linear, and either fixed or continuously increasing. Studies utilizing gain displacements were excluded. Participants must have seen a representation of their hands in the form of a virtual hand, finger, or fingertip depicted as a geometric shape; studies focusing on unseen hands, other body parts, or tools were excluded.

Finally, included studies were required to measure and precisely define detection thresholds for positional shifts, with estimated values clearly stated and described.

### 2.3 Information sources and search strategy

Literature searches were conducted using two databases: Scopus and Web of Science. The most recent search was completed on May 15, 2024. Research areas included Computer Science, Engineering, Neurology, Psychiatry, Neuroscience, and Psychology, with searches performed on titles, abstracts, and keywords.

Given the technological focus of this review, terms like *proprioception* and *multisensory integration* were excluded to avoid limiting the search to medical literature. Instead, broader, general terms were selected to maximize the retrieval of relevant studies. Search terms were grouped into five categories: *virtual reality*, *detection threshold*, *hand*, *shift*, and *participants*. The search string is listed in full in the **Supplementary Materials**.

### 2.4 Data items

Data items were chosen to answer the following research questions:

1. Does the direction of positional offsets influence the detection?
*Direction of positional offsets* refers to the specific spatial relationship between virtual and real hands. In relation to real hands location, the virtual hands might be shifted in a horizontal manner (X-axis), vertical manner (Y-axis) or sagittal manner (Z-axis).
2. Is the sensitivity to positional offsets influenced by their character in time?
  The magnitude of displayed positional offsets might remain temporally constant or evolve (i.e. increase or decrease). This attribute of positional offsets is expressed as *character in time*.
3. Does the distance of the hand and the direction of hand reach influence the detection of positional offsets?
  The first part of this research question asks whether the distance between the hand and the body influences the ability to detect positional offsets of virtual hands. This systematic review also investigates whether the position of the target and thus the direction of a moving hand influences the offset detection.
4. Does the depiction of the hand in virtual reality influence the detection of positional offsets?
  The virtual extremities might be depicted as realistic hand-shaped objects or as abstract objects lacking the resemblance to real hands.
5. Can a distraction influence the detection threshold of positional offsets?
  For the purpose of this systematic review, *distractions* refer to additional sensory signals that tend to either mislead or divert attention.
6. Are detection thresholds of positional offsets influenced by participant’s gender?
7. Does handedness influence the detection of positional offsets?

## 3 Results

**Figure 1:**
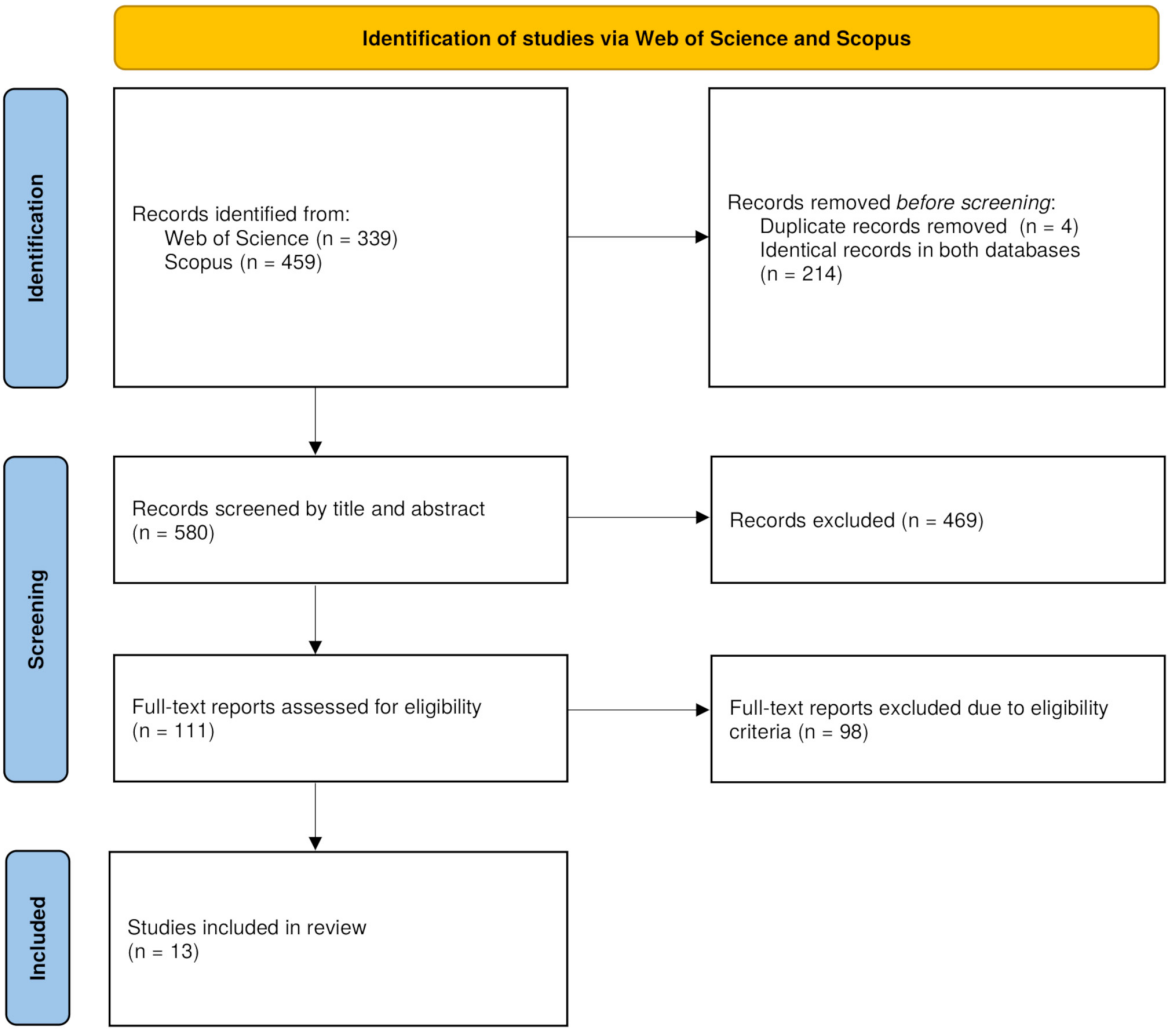
PRISMA diagram (18).

After the database search and records screening, 13 studies were identified as relevant based on the eligibility criteria, as illustrated in the PRISMA flow diagram (see **Figure 1**). These studies employed various methodologies and terminology; related terms and concepts are explained in **Table 1**. Basic information about the included studies and their samples is summarized in **Table 2**, while technical characteristics and paradigm details are outlined in **Tables 3**, **4**, respectively. **Table 5** provides a summary of the results, focusing on the extent of offset detection. For clarity, the term *higher sensitivity* is used to describe a smaller detection threshold in outcome comparisons. The text below offers a concise summary of the findings, addressing the research questions outlined earlier.

**Table 1:** Terms and concepts.

Term/concept category	Term/concept	Description
Positional offset category (concepts)	fixed offset	pre-defined difference between VH and PH
	i) fixed angular offset	pre-defined angular difference between VH and PH
	ii) fixed linear offset	pre-defined length difference between VH and PH
	iia) fixed linear (absolute) offset	difference between VH and PH present from the beginning of the trial, constant until a specified event/end of the trial
	iib) fixed linear (relative) offset	offset increasing to the pre-defined length difference between VH and PH
	continuously increasing offset	difference between VH and PH continuously changing in time, no pre-defined offset
	i) continuously increasing angular offset	angular difference between VH and PH continuously changing in time, no pre-defined offset
	ii) continuously increasing linear offset	length difference between VH and PH continuously changing in time, no pre-defined offset
Direction of positional offsets	X-axis	horizontal offsets (in relation to forward direction: leftward, rightwards offsets)
	Y-axis	vertical offsets (in relation to forward direction: upward, downward offsets)
	Z-axis	sagittal offsets (in relation to forward direction: offsets closer, farther from the body)
Sampling method	method of limits	series of ascending and descending trials, DT=average of transition points (41)
	method of constant stimuli	same stimuli used repeatedly, DT anticipated within the range of stimuli (41)
	staircase procedure	sequence of stimuli increasing/decreasing in time, direction reverses after the change of the answer, DT=average of transition points (41)
Detection threshold definition	just-noticeable difference	minimum difference between two stimuli for considering reliable detectability (with defined proportion of correct answers) (39)
	point of subjective equality	minimum difference between two stimuli recognizable as distinct with a 50% accuracy rate (39)

DT, detection threshold; PH, physical hand; VH, virtual hand.

**Table 2:** Included studies and sample information.

Study	Sample size; gender; handedness	Age
Burns et al. (2006) (21)	N=40; m=19, f=21; RH=40	M, SD not reported
Deligiannidis et al. (2009) (19)	N=8; m=4, f=4; RH=4, LH=4	18-36 (M, SD not reported)
Lee et al. (2015) (27)	N=6; m=6; RH=6	22-32 (M, SD not reported)
Zenner and Kruger (2019) (20)	N=12; m=6, f=6; RH=11, LH=1	M=28 (SD not reported)
Gonzalez and Follmer (2019) (24)	N=14; m=6, f=8; RH=14	M=27, SD=5
Benda et al. (2020) (22)	N=19; m=13, f=6; RH=16, LH=3	M=23.5 (SD not reported)
Ogawa et al. (2021) (26)	N=17; m=14, f=3; RH=16, LH=1	M=24.5, SD=4.4
Zenner et al. (2021) (25)	N=15; m=8, f=7; handedness not reported	M=25.5, SD=3.5
Clarence et al. (2022) (23)	N=22; m=13, f=9; RH=22	M=26.1, SD=7.9
Kohm et al. (2022) (32)	N=20; m=14, f=5, non-conforming/nonbinary=1; RH=18, LH=2	18-33 (M, SD not reported)
Ogawa et al. (2023) (28)	N=22; m=11, f=11; RH=21, LH=1	M=22.6, SD=0.9
Yang et al. (2023) (29)	N=19; m=12, f=7; RH=19	M=22.2, SD=1.1
Zenner et al. (2023) (42)	N=20; m=11, f=9; RH=20	M=27, SD=10

F, female; LH, left-handed; m, male; M, mean; N, sample size; RH, right-handed; SD, standard deviation.

**Table 3:** Technical characteristics of the hardware and software used in the studies.

Study	HMD	Hand tracking	Software	Technical comments
Burns et al. (2006) (21)	Virtual Research Systems V8	Outside-In tracking: 3rdTech Hiball 3000 (optical tracking device)	not reported	6 datasets lost due to SW malfunction
Deligiannidis et al. (2009) (19)	Virtual Research Systems V8	Outside-In tracking: Flock of Birds (pulsed DC magnetic technology)	not reported	
Lee et al. (2015) (27)	Oculus Rift CV1	Outside-In tracking: VICON MOCAP system	OpenGL	
Zenner and Kruger (2019) (20)	HTC Vive	Outside-In tracking: HTC Vive Tracker - lighthouse tracking + IMU	Unity 3D	
Gonzalez and Follmer (2019)	Oculus Rift CV1	Outside-In tracking: OptiTrack Prime 13 (1,3 MP, 240 Hz)	Unity 3D	
Benda et al. (2020) (22)	Oculus Rift CV1	Inside-Out tracking: Oculus Touch controllers	Unity 3D	Calibration for each participant
Ogawa et al. (2021) (26)	Oculus Rift CV1	Inside-Out tracking: Leap Motion Controller	Unity 3D	KONICA MINOLTA PULSOX- Lite (Heartbeat separate test)
Zenner et al. (2021) (25)	HTC Vive Pro Eye	Outside-In tracking: HTC Vive Tracker (v2018) + SteamVR base stations 2	Unity 3D	
Clarence et al. (2022) (23)	HTC Vive Pro	Outside-In tracking: HTC Vive Tracker, Vive Base Stations	Unity 3D	
Kohm et al. (2022) (32)	Meta Quest 1	Inside-Out tracking based on hands: Built-in hand tracking system	not reported	HMD forward gaze vector used to approximate gaze direction. The only HMD in the review used without a cable.
Ogawa et al. (2023) (28)	Meta Quest 2	Inside-Out tracking based on hands: Built-in hand tracking system	Unity 3D	Control of the electrical stimulator via serial communication from Unity - however, reaction time is not so important here, the most influential is how authentic the noise was.
Yang et al. (2023) (29)	Oculus Rift CV1	Outside-In tracking: OptiTrack Prime 13 (1,3 MP, 240 Hz) - 4 cameras	Unity 3D	
Zenner et al. (2023) (42)	HTC Vive Pro Eye	Outside-In tracking: HTC Vive Tracker, unspecified spatial boundary technique for saccade onset detection	not reported	

**Table 4:** Studies characteristics.

Study	Study characteristics										
	Detection threshold task	Extremity tested, visual representation	Positional offset category (PH vs. VH)	Direction of positional offsets (PH vs. VH)	Magnitude of positional offsets (PH vs. VH)	Reaching distance	Sampling method	Detection threshold definition	Haptics, distraction	Conditions	Embodiment/presence measure
Burns et al. (2006) (21)	game similar to Hasbro's Simon	right hand (dominant)	continuously increasing angular offset	X-axis (L, R), Y-axis (U, D)	0–60°; 0.46°/s; shoulder being the centre of the circle	arm stretched out during the experiment, the distance between the hand and the shoulder was measured	partial MoL (ascending series only)	position discrepancy at time of report - reaction time × hand speed	0	unprimed threshold (not primed to expect the visual-proprioceptive discrepancy) = 1 shift direction (L); primed threshold (after priming to expect the discrepancy) = 4 shift directions (L, R, U, D)	0
Deligiannidis et al. (2009) (19)	single-interval Yes-No task after passive movement on a 3x3 array (1-AFC)	index fingertip of the dominant hand (1.5 cm² cubes)	fixed linear (absolute) offset	X-axis (L, R), Z-axis (F, C)	0, 2, 3, 4, 5 cm (17 locations for each square)	3x3 array; distance between the participant's body and the edge of the desk unlisted; distance between the edge of the desk and the closest row of the workspace=26 cm; distance between centres of the closest and the farthest row = 22.8 cm	MCS (square (of the 3x3 array) + finger position on the square randomly selected)	75% hit rate modified by a signal detection analysis (for taking into account the number of false alarms)	0	9 fields (3x3 array), 4 shift directions	0
Lee et al. (2015) (27)	sliding on the cylinder surface, haptic/no haptic feedback while touching the cylinder, 2-AFC: discriminating between 2 positions (2 spheres = false, true positions of the index fingertip)	index fingertip of the right (dominant) hand (sphere)	fixed linear (absolute) offset	false sphere in the circle with the true sphere being the center of the circle	radial distances (between false and true spheres)=1.5–7.5 cm; by 1.5 cm	2 spheres in front of the participant; reaching distance = 30–50 cm	MCS (randomly in the circle (the true index finger being the centre of the circle))	JND=75% correct answers	cutaneous haptic device	with/without haptic feedback	0
Zenner and Kruger (2019) (20)	target-touching task (2-AFC)	dominant hand	fixed angular offset	X-axis (L, R), Y-axis (U, D)	-14°–14° (15 angles); by 2°	start location (warp origin) = 30 cm beneath, 30 cm in front of the head, target = 40 cm in front of the start location	MCS (randomly generated discrepancies)	discrimination thresholds = psychometric function intersecting 75% probability (R, U) and 25% probability (L, D) of detection	auditory+vibrotactile distraction, visual distraction+increased cognitive load	scenarios: no distraction, audio-vibrotactile distraction, visual-cognitive distraction; 4 shift directions	SUS (43); ratings: body ownership, hand control
Gonzalez and Follmer (2019) (24)	target acquisition task (1-AFC)	dominant, non-dominant hand	fixed angular offset	Y-axis (U, D)	-24°, -18°, -12°, -6°, 6°, 12°, 18°, 24° (8 angles; shoulder being the centre of the circle, not the warp origin)	distance between the shoulder and the target derived from the arms’s length; target = 17 cm above the desk, 50 cm in front of the starting point	MCS (randomly generated discrepancies)	PSE	passive haptics (physical props = 2 spheres)	bimanual conditions (same-directional, opposite-directional), single-hand conditions; 2 shift directions	0
Benda et al. (2020) (22)	target-touching task (2-AFC)	right hand	fixed linear (absolute) offset	X-axis (L, R), Y-axis (U, D), Z-axis (F, C)	0–24 cm; by 3 cm	distance between the chest and the centre of the target group = 24 cm	MCS (randomly generated discrepancies)	PSE	0	6 shift directions	0
Ogawa et al. (2021) (26)	target-touching task (1-AFC)	right hand/right index fingertip (abstract sphere)	fixed linear (relative) offset	X-axis (L, R)	ascending staircase (0–10 cm) + descending staircase (10–0 cm); staircase by 1.5 cm	starting point = in front of the participant's head, 35 cm distance from the HMD, height depends on the arm's length; target = 20 cm in front of the starting point, 10 cm below the starting point; distance of the starting point and the target from the body midline = 10 cm	interleaved staircase procedure	average value of the thresholds of the ascending and descending series for each condition	0	types of staircases: 2 levels of avatar appearance (realistic hand/abstract sphere) x 2 shift directions x 2 initial shifts (10 cm/0 cm)	AEQ (44), self-localization task
Zenner et al. (2021) (25)	target-touching task (1-AFC)	dominant hand	fixed linear (absolute) offset (instantaneously increasing after blinks, by staircase), continuously increasing linear offset	X-axis (R), Y-axis (D)	ascending staircase (0–8 cm) + descending staircase (8–0 cm); staircase by 0.8 cm	start location = 30 cm beneath and 25 cm in front of the head, target = 40 cm in front of the start location	interleaved staircase procedure	average value of the thresholds of the ascending and descending series for each condition	blinks	types of staircases: 4 hand redirection techniques (1. blink-suppression hand redirection BSHR+0% (instantaneous redirections up to % of the DT from (20)), 2. BSHR+50%, 3. BSHR+100%, 4. Cheng et al.'s method (45) (unlimited continuous redirection)) x 2 shift directions x 2 initial shifts (8 cm/0 cm)	SUS (43)
Clarence et al. (2022) (23)	target-touching task (2-AFC)	right hand (dominant)	fixed angular offset	X-axis (L, R); L=counterclockwise, R=clockwise	each target location (from the warp origin): 0°; clockwise, counterclockwise (6°, 12°, 18°, 24°, 30°)	cylinders placed on a desk (8 target directions: 0°, 45°, 90°, 135°, 180°, 225°, 270°, 315°); cylinder 6 (centre of the semicircle) = 20 cm away from the body midline; reaching distance = 30 cm	MCS (stimuli in randomised order)	PSE	passive haptics (physical props = 6 cylinders, cylinders 1-5 form a semicircle, with cylinder 6 being the centre of the semicircle)	8 reaching directions, 2 shift directions (clockwise, counterclockwise)	self-localization task
Kohm et al. (2022) (32)	block placing tasks (2-AFC) in 4 weeks of intensive VRE presence	dominant hand	fixed linear (absolute) offset	X-axis (L, R), Z-axis (F, C), intermediate steps: F-R, C-R, C-L, F-L; all directions closer to the body considered as negative discrepancies (RH: L, C-L, C, C-R; LH: R, C-R, C, C-L), all directions farther from body considered as positive discrepancies (RH: R, F-R, F, F-L; LH: L, F-L, F, F-R)	0–14 cm; by 2 cm	distances unlisted, 3 virtual blocks placed on the table semi-randomly	MCS (randomly generated discrepancies)	PSE	0	4 sessions, 2 tasks in each trial (1. with offset, 2. with no offset)	0
Ogawa et al. (2023) (28)	target touching task (1-AFC)	right hand	fixed angular offset	X-axis (L, R)	-15°–15° (11 angles); by 3°	start location = 30 cm beneath, 30 cm in front of the HMD; target = 40 cm in front of the start location	MCS (randomly generated discrepancies)	PSE	noisy tendon (biceps, triceps brachii muscles) electrical stimulation (leading to the decrease of the impact of proprioception)	with/without tendon electrical stimulation, 2 shift directions	VEQ (46), IPQ (47)
Yang et al. (2023) (29)	reach-to-grasp, reach-to-place task (pseudo-2-AFC)	right hand (dominant)	fixed linear (relative) offset	X-axis	0–20 cm; by 4 cm	initial distance of the virtual object (and the physical prop) from the centre of the desk = 20 cm to the left; reach-to-grasp task: distances unlisted; reach-to-place task: moving distance for the virtual rod = 20 cm to the right, moving distance for the physical rod = 20 cm+offset magnitude	MCS (randomly generated discrepancies)	PSE	passive haptics (physical prop=rod)	3 redirections (reach-to-grasp, reach-to-place, combination)	0
Zenner et al. (2023) (42)	distraction by a visual target (leading to a saccade) followed by a VH shift (1-AFC)	right hand (dominant)	fixed linear (absolute) offset (increasing/decreasing by staircase)	X-axis (L, R)	ascending staircase (from 0 cm) + descending staircase (from 6 cm); staircase by 1 mm (down) and 3 mm (up)	hand was on the same level as the desk; distance between the participant's body and the hand unlisted	interleaved staircase procedure	75%-correct DT	saccades	types of staircases: 5 saccade-VH angles (α = 0°, 45°, 90°, 135°, 180°) x 2 shift directions x 2 initial shifts (6 cm/0 cm)	0

1-AFC, one-alternative forced-choice task; 2-AFC, two-alternative forced choice task; AEQ, Avatar embodiment questionnaire (44); BSHR, blink-suppressed hand redirection; C, shift closer to the body; D, downward shift; DT, detection threshold; F, shift farther from the body; IPQ, Igroup Presence Questionnaire (47); JND, just-noticeable difference; L, leftward shift; LH, left-handed; MCS, method of constant stimuli; MoL, method of limits; PH, physical hand; PSE, point of subjective equality; R, rightward shift; RH, right-handed; SUS, Slater-Usoh-Steed Presence Questionnaire (43); U, upward shift; VEQ, Virtual Embodiment Questionnaire (46); VH, virtual hand; VRE, virtual reality environment.

**Table 5:** Results.

Study	Results outcome (M, [95% CI]/SD if reported)
Burns et al. (2006) (21)	Unprimed trials: DT=45.4° ( $\tilde{4}2$ cm), primed trials: DT=19.1° ( $\tilde{1}9$ cm), significant difference, assessed as inconclusive. No statistically significant differences for different directions.
Deligiannidis et al. (2009) (19)	Displacements *<* 5 cm not reliably detected. Sensitivity to 5 cm shifts: a function of shift direction and handedness. Higher sensitivity to R than L (significant difference only in the first row), C than F (significant difference only in the first row), X-axis than Z-axis. The highest sensitivity in the nearest row. Sensitivity to R, C decreased with distance, sensitivity to L, F increased with distance. No main effect of the rows. Main effect of the columns, the highest sensitivity in the centre column. RH more sensitive than LH, marginally significant.
Lee et al. (2015) (27)	DT (no-haptic feedback) = 5.2 cm, DT (false haptic feedback) = 6.2 cm, DT (true haptic feedback) ~3.3 cm. False haptic feedback led to the DT increase, true haptic feedback led to the DT decrease.
Zenner and Kruger (2019) (20)	DT (no-distraction): X-axis shifts = 8.2° (R=3.8°, L=4.4°), Y-axis shifts = 8.9° (U=4.5°, D=4.4°). DT (audio-vibrotactile distraction): X-axis shifts = 7.9° (R=2.3°, L=5.6°), Y-axis shifts = 9.3° (U=4.6°, D=4.7°). DT (visual-cognitive distraction): X-axis shifts: 10.1° (R=2.9°, L=7.2°), Y-axis shifts: 9.9° (U=4.7°, D=5.3°). The overall R =3.1°, L = 5.8°, U = 4.6°, D = 4.8°. Higher overall sensitivity to R than L and U than D. In audio-vibrotactile or visual-cognitive distraction condition, most sensitive to R, least sensitive to L. Higher sensitivity to X-axis than Y-axis (no-distraction), X-axis than Y-axis (audio-vibrotactile), Y-axis than X-axis (visual-cognitive). Distraction did not reliably increase DT. DTs were not tested for statistically significant differences.
Gonzalez and Follmer (2019) (24)	DT (single-hand retargeting) for right hand: D=-16.4° [-19.0, -14.1], U=17.1° [14.1, 20.9]; left hand: D=-16.2° [-18.9,-13.8], U=18.5° [15.2, 22.0]. DT (same-directional bimanual retargeting) for right hand: D=-19.5° [-21.8, -17.1], U=21.4° [17.7, 26.9]. DT (opposite-directional bimanual retargeting) for right hand: D=-12.3° [-15.0, -9.3], U=14.3° [11.7, 17.2]. Higher sensitivity to D than U. Higher overall sensitivity to right hand. Higher sensitivity to opposite-directional than same-direction bimanual retargeting.
Benda et al. (2020) (22)	DT: R=9.4 [8.5, 10.2] cm, L=10.3 [9.3, 11.3] cm, U=12.8 [11.7, 14.1] cm, D=13.4 [12.3, 14.5] cm, F=13.3 [12.1, 14.4] cm, C=7.8 [7.1, 8.6] cm. Higher sensitivity to R than L, U than D, C than F. Sensitivity between axes: X-axis *>* Z-axis *>* Y-axis. Based on significance, 3 groups: C, R+L, U+D+F; significant differences between groups, not between members of groups. Within axes, significant difference only in Z-axis.
Ogawa et al. (2021) (26)	L: significantly higher sensitivity for abstract avatars ( $\text{DT}\overset{3}{\sim }.4$ cm) than realistic avatars ( $\text{DT}\overset{4}{\sim }.5$ cm), R: not significantly higher sensitivity for realistic avatars than abstract avatars (both $\text{DTs}\overset{5}{\sim }$ cm). Higher sensitivity to L than R, significant only in abstract avatars.
Zenner et al. (2021) (25)	DT (BSHR+0%): R=2.7 (SD=1.3) cm, D=3.8 (SD=1.1) cm; DT (BSHR+50%): R=3.6 (SD=1.3) cm, D=4.9 (SD=1.5) cm; DT (BSHR+100%): R=4.3 (SD=1.5) cm, D=5.4 (SD=1.2) cm; DT (Cheng et al.’s method): R=5.8 (SD=2.0) cm, D=5.6 (SD=2.1) cm. R (arranged from the highest sensitivity): BSHR+0% *>* BSHR+50% *>* BSHR+100% *>* Cheng et al.’s method, significant differences between all techniques. D (arranged from the highest sensitivity): BSHR+0% *>* BSHR+50% *>* BSHR+100% *>* Cheng et al.’s method, significant difference between BSHR+0% and BSHR+50% and BSHR+0% and BSHR+100%. BSHR+0%, BSHR+50%, BSHR+100% = higher sensitivity to R than D. Cheng et al’s method = slightly higher sensitivity to D than R.
Clarence et al. (2022) (23)	DT (overall) = 16.1° [15.8, 16.6], R = 16.8° [16.2, 17.4], L = 15.6° [15.1, 16.2]. Higher sensitivity when reaching towards the body: DT (reaching towards the body)=15.7° [15.0, 16.3], DT (reaching away from the body)=16.5° [16.0, 16.9], significant difference. Gender: no effect on discrepancy detection.
Kohm et al. (2022) (32)	Significant differences in DT between sessions (except 1. session: significant difference only between 1. and 2. session). The overall DT: -0.7 [-1.5, 0.2] cm (1. session), -2.1 [-3, -1.2] cm (2. session), 0.5 [-0.4, 1.3] cm (3. session), -0.8 [-1.7, -0.1] cm (4. session). Participants equally sensitive to positive and negative offsets. Sensitivity to offsets did not change over time (no significant differences between slopes).
Ogawa et al. (2023) (28)	X-axis DT range (with ES): 20.5° (SD = 7.9), X-axis DT range (without ES): 19.2° (SD = 7.1), significant effect of ES. Male DT range (with ES): 15.4° (SD = 6.1), DT range (without ES): 15.2° (SD = 5.6). Female DT range (with ES): 25.6° (SD = 5.9), DT range (without ES): 23.1° (SD = 6.2). Within gender, significant effect of ES only in women. DT overall significantly larger in women.
Yang et al. (2023) (29)	Overall reach-to-grasp: DT=11.8 [9.9, 13.7] cm, reach-to-place: DT=16.0 [14.4, 17.5] cm, combination: DT=12.4 [10.6, 14.2] cm. Higher sensitivity to reach-to-grasp than reach-to-place redirection. Male reach-to-grasp: DT=13 cm, reach-to-place: DT=19 cm, combination: DT=13.4 cm. Female reach-to-grasp: DT=10.1 cm, reach-to-place: DT=14.7 cm, combination: DT=10.7 cm. Women more sensitive to redirection than men.
Zenner et al. (2023) (42)	Saccade and hand offset in opposite directions (*α>*90): shifts unnoticed in the cm range. Saccade and hand offset in the same direction (*α ≤*90): shifts unnoticed in the mm range. DTs significantly increase with greater saccade-VH angles.

BSHR, blink-suppressed hand redirection; C, shift closer to the body; CI, confidence interval; D, downward shift; DT, detection threshold; ES, electrical stimulation; F, shift farther from the body; JND, just-noticeable difference; L, leftward shift; LH, left-handed; M, mean; R, rightward shift; RH, right-handed; SD, standard deviation; U, upward shift; VH, virtual hand.

### 3.1 Sample sizes and demographic characteristics of included studies

Most of the reviewed studies (ten in total) included 20 or fewer participants. Two of the assessed studies included 22 participants. One study recruited 40 participants.

The number of male participants exceeded the number of female participants in a total of seven studies. In two reviewed studies, the pattern inverted with a predominance of female participants. In three of the reviewed studies, the number of male and female participants corresponded. One study was performed only in male participants.

Six studies were performed only in right-handed participants. Other six studies recruited both right- and left-handed participants. In five of these studies, right-handed participants prevailed, only one study was performed on corresponding numbers of left- and right-handed participants. One study did not report handedness.

In nine studies, the mean age of the participants falls under the age group 20-30, standard deviation is reported in seven of these studies. Although mere age intervals were reported in three studies, they indicate that the predominant age group of the participants will not significantly differ from other studies. One study did not report the participants’ age.

The demographic characteristics of samples in the eligible studies are summarized in **Table 2**.

### 3.2 Research question 1: does the direction of positional offsets influence the detection?

Shifts can be categorized based on the axis related to the hand-target trajectory: X-axis (right-left), Y-axis (up-down), and Z-axis (farther-closer). In studies examining the X-axis, participants were more sensitive to rightward shifts in two studies (19, 20), with statistical significance reported in one (19). However, two other studies found no significant differences within the X-axis (21, 22). One study noted greater noticeability of leftward shifts but did not report statistical significance (23). Of note, the overwhelming majority of the subjects were right-handed.

For the Y-axis, studies reported mixed findings: one study found higher sensitivity to upward shifts (20), and another to downward shifts (24), neither reporting p-values. Two other studies observed no significant differences along the Y-axis (21, 22).

Shifts towards the body were detected more easily than those away from the body in two studies (19, 22). Comparatively, participants consistently showed higher sensitivity to shifts along the X-axis than those along the Y-axis (20, 22, 25) and Z-axis (19, 22). One study outlined an order of sensitivity from highest to lowest as X-axis, Z-axis, then Y-axis (22).

### 3.3 Research question 2: is the sensitivity to positional offsets influenced by their character in time?

Shifts can be categorized based on their temporal characteristics into fixed and continuously increasing types. Zenner et al. (25) explored two specific forms of shifts: instantaneously increasing shifts (fixed shifts that increase after blinks) and continuously increasing shifts. The study found that participants were most sensitive to instantaneously increasing shifts. Detection thresholds significantly rose when continuous shifts were introduced. Furthermore, continuous shifts, without preliminary exposure to fixed shifts, exhibited the lowest detection sensitivity.

### 3.4 Research question 3: does the distance of the hand and the direction of hand reach influence the detection of positional offsets?

This question was explored in two studies. Deligiannidis et al. (19) analyzed shifts within a workspace divided into three rows at varying distances from the participant and three columns along the sagittal plane. Detection was most effective in the row closest to the participant, and in the median plane. However, statistical analysis showed only a significant effect of the column.

In a second study by Clarence et al. (23), participants interacted with eight targets at different distances. The results showed significantly higher sensitivity to discrepancies when reaching towards the body compared to reaching away from the body.

### 3.5 Research question 4: does the depiction of the hand in virtual reality influence the detection of positional offsets?

A single study investigated the effect of virtual hand depiction on the sensitivity to positional shifts (26). The findings indicated that the detection threshold for positional shifts was significantly lower when abstract objects were used compared to realistic virtual hands, but this was only the case for leftward shifts. For rightward shifts, there was no significant difference in detection thresholds between the realistic and abstract avatars.

### 3.6 Research question 5: can a distraction influence the detection threshold of positional offsets?

Three studies explored the impact of distractions on detection thresholds. In the first study, Lee et al. (27) found that false haptic feedback on an index finger increased the *just noticeable difference*, although the statistical significance was not reported. The second study by Zenner et al. (20) used audio-vibrational distractions mimicking a flying bee, along with visual distractions coupled with a heightened cognitive load, reporting only minor differences between distracted and undistracted conditions. In the third study, Ogawa et al. (28) applied noisy tendon electrical stimulation, which significantly increased the detection threshold.

### 3.7 Research question 6: are detection thresholds of positional offsets influenced by participant’s gender?

Three studies examined the potential effect of participant gender on detection thresholds, yielding mixed results. Clarence et al. (23) found no significant differences between genders. Conversely, Ogawa et al. (28) reported that men were significantly more sensitive to displacements. Meanwhile, Yang et al. (29) observed higher sensitivity among women, although they did not report a p-value for this finding.

### 3.8 Research question 7: does handedness influence the detection of positional offsets?

In a single study (19), right-handed subjects were more sensitive than left-handed subjects. According to the authors, the difference was ‘marginally significant’.

### 3.9 Hand tracking accuracy and technological considerations

The accuracy of hand tracking in VR devices is a factor that significantly influences the assessment of results. Hand tracking systems vary between *inside-out* tracking, where sensors are built into the headset facing outward, and *outside-in* tracking, which uses external sensors directed towards the headset. Insideout devices can have maximum measurement deviations exceeding 12.0 mm, whereas outside-in systems generally show measurement errors as low as 0.4 mm (30). Among the studies reviewed, nine employed outside-in tracking systems, in contrast to four using the less accurate inside-out tracking. Notably, two of these studies utilized the Meta Quest’s built-in hand tracking, potentially introducing an additional positional error of up to 1.1 cm in fingertip location (31), significantly impacting results as demonstrated by Kohm et al., where positional offset results ranged from -2.1 to 0.5 cm (32).

The HTC Vive Tracker, an outside-in tracking system, was used in four studies, where it was noted that the maximum absolute distance error in a constrained area could reach up to 14.9 mm (33).

Various technological complications impacting data integrity and results accuracy were reported. Burns et al. experienced the loss of six datasets due to software malfunctions (21). Ogawa et al. attributed biases in their results to the absolute error in hand position estimation by the inside-out Leap Motion Controller (26). Kohm et al. relied solely on the headset’s gyroscopes to approximate gaze direction, operating the HMD without a tether, which could affect immersion and outcomes (32). Clarence et al. speculated that the VR setup and specific methods of hand redirection might influence the reported detection limits (23).

Further, technological issues extended beyond VR tracking. Ogawa et al. observed an unintended hint given to participants estimating their pulse due to the pressure from a pulse oximeter, which might have influenced their actual results (26). A second study from the same laboratory reported varied results based on gender, speculating that the increased impact of electrical stimulation on women could explain differences in offset tolerance, without accounting for potential variations in VR experience by gender (28).

## 4 Discussion

The purpose of this systematic review was to explore the current knowledge about the threshold of the virtual hand *positional offset*, i.e. the limits in which the user perceives the virtual hand as their own and factors influencing it, including the effect of various hardware setups. *Matching* between actual and virtual limbs is, together with optimal *self-presentation*, a crucial aspect of VR immersion and, therefore, presence. Understanding offset detection limits may help to create more immersive VR interventions and set a baseline for research on body ownership in various clinical populations, which could open new avenues for diagnostic VR paradigms.

The most important finding of this review is that reported detection thresholds for a positional discrepancy between a virtual and a physical hand are very heterogeneous and depend on several factors. It seems that even in healthy individuals, awareness of physical hand boundaries is not rigid, depends on several factors, and can probably be successfully manipulated.

Regarding absolute values, the threshold for detecting positional offsets ranges from a few millimeters to 42 cm for linear shifts and from 2° to 45°for angular offsets. However, these values likely overestimate true inter-individual variability, as they are influenced by multiple factors, including experimental setups, multisensory integration mechanisms (34), individual participant characteristics, and hardware specifications.

Firstly, misalignment detection seems to be influenced by the direction of discrepancies and their changes over time. Sensitivity appears to be highest within the X-axis (horizontal left-right), followed by the Z-axis (horizontal farther-closer) and the Y-axis (vertical). Additionally, continuously increasing shifts were found to be less detectable than those that increase instantaneously (25). Additionally, higher sensitivity to positional offsets was found for a dominant hand. Furthermore, shifts closer to the body are more noticeable than those farther away. Consequently, there is greater leeway for matching between the virtual and real limb when reaching outward. This implies that requirements for *matching* should be more stringent in experimental paradigms or interventions focusing on actions close to the body, as compared to applications like VR fitness or VR exercise programs, that were lately utilized to improve quality of life in elderly (35) or in depression and anxiety (36).

The second group of influencing factors underscores the well-established role of multisensory integration in self-location (37). These factors should be considered when designing paradigms that explore body ownership or agency, as well as in VR interventions. Visually, a more accurate representation of a human hand allowed for a greater tolerance to positional offsets compared to abstract shapes. However, the role of haptic feedback is more complex. Intriguingly, corresponding haptic feedback reduced the detection threshold—meaning it necessitated a more precise match between the hand and its virtual representation. Conversely, sensory distractions such as false haptic feedback and noisy tendon electrical stimulation seemed to increase the threshold for offset detection. Unfortunately, the role of passive haptics remains unclear, as it was implemented in only three studies. It is plausible to hypothesize that while the use of physical props can enhance VR presence (38), their impact might be similar to that of matching haptic feedback, thereby heightening the matching requirements. While multisensory integration is important in the neuroscientific understanding of this topic, it was not a primary focus in the reviewed studies. Therefore, the correlation between the characteristics of positional offset detection and multisensory integration should be considered putative rather than established.

The third set of variables influencing offset sensitivity relates to inter-individual variability. Unfortunately, the effect of gender remains unclear due to ambiguous results. Similarly, the influences of age and VR experience cannot be clearly established; only a single study explored these factors, finding that while age and VR experience have a statistically significant influence, they account for only minimal variability among individuals (23). Concerning handedness, right-handed individuals appeared to be more sensitive to positional shifts than left-handed individuals. However, the representation of left-handed individuals in studies was exceptionally low, ranging from 1 to 4, and they constituted a negligible fraction of participants, if included at all (in 5 out of 13 studies). Consequently, drawing any meaningful conclusions about the influence of handedness is challenging.

An interesting finding was reported by Kohm et al., suggesting that sensitivity to positional offsets seems to be stable over time (32). This stability implies that the sensitivity to detecting misalignment between virtual and real hands may be a trait characteristic of an individual. This aligns with observations of an aberrant sense of body ownership and agency in certain neuropsychiatric disorders, notably schizophrenia (12). If confirmed, and if it varies with the clinical state, this observation could pave the way for using positional offset detection as a diagnostic tool.

Other factors might also influence the detection of positional offsets, though their impacts have not been thoroughly investigated in the reviewed studies. One such example is the complexity of a task. Studies generally employed simple paradigms such as target-touching tasks, grasping and placing objects, or following along virtual shapes. Only a single study introduced a more complex setup: Burns et al. required participants to engage in a game demanding high concentration, which could have affected their final detection thresholds—these were notably higher compared to other studies (21). It is important to mention that the applied offsets were constantly increasing, and therefore more challenging to detect (25).

An essential factor to consider is the accuracy and technological capabilities of VR equipment, which have dramatically improved over time. The evolution from the early 2000s’ Virtual Research Systems V8 HMD, featuring a resolution of 640x480 pixels per eye and a 60° field of view, to the more recent Meta Quest 2 HMD, with its 1832x1920 pixels per eye and 90°field of view, underscores the rapid technological progress in this field. This advancement is detailed in **Table 6**, summarizing the technological parameters of HMDs used in the reviewed studies. Results should be evaluated within this context of continual technological improvement.

**Table 6:** HMD comparison.

HMD	Year	Resolution	FoV	Frequency	Cable required	Comment
Virtual Research Systems V8	Early 1990s	1280x480 (640x480 pe)	60°	60 Hz (30 Hz pe)	Yes	Originally cost approximately $13 000
Oculus Rift CV1	2016	2400x1080 (1080x1200 pe)	90°	90 Hz	Yes	
HTC Vive	2016	2160×1200 (1080×1200 pe)	∼110°	90 Hz	Yes	Tracking system Lighthouse (2 base stations emitting pulsed IR lasers)
HTC Vive Pro Eye	2019	2880x1600 (1440×1600 pe)	∼110°	90 Hz	Yes	Additional built-in eye tracking, higher resolution
HTC Vive Pro	2018	2880x1600 (1440×1600 pe)	∼110°	90 Hz	Yes	Without eye tracking
Meta Quest 1	2019	2880x1600 (1440 × 1600 pe)	∼92°	72 Hz	No	Inside-Out tracking
Meta Quest 2	2020	3664x1920 (1832x1920 pe)	∼89°	72-120 Hz	No (used anyway)	Inside-Out tracking. Frequency based on application. Absolute positional error is found to amount to 1.1 cm in average, average temporal delay of 45.0 ms (31).

FoV, Field of Vision; pe, per eye.

### 4.1 Limitations of the reviewed studies

Several limitations within the presented studies may have impacted the quality and, consequently, the reliability of the results.

A notable portion of the source material comprises conference papers, with 10 out of 13 research studies reported in this format. However, the majority of these conference papers (7 out of 10) underwent peer review. This aspect adds a layer of credibility, though the consistency of review processes for conference papers can vary compared to journal publications.

A fundamental issue is the lack of a uniform definition of the offset detection threshold across studies. Most research employed the concept of *the point of subjective equality*, defined as the minimum difference between two stimuli recognizable as distinct with a 50% accuracy rate (39). However, not all reviewed studies adhered to this definition, introducing variability in how offset detection thresholds were quantified.

From a methodological perspective, the sample sizes in most studies were small, with only two studies conducting a power analysis (26, 28). Additionally, in several studies examining differences between various conditions, p-values were insufficiently reported. Reporting on demographic variables was also often incomplete; key characteristics such as handedness or age were missing in seven studies. Predominantly right-handed subjects were recruited, and most experiments focused on the dominant hand. The lack of assessment concerning technological familiarity due to previous VR experience might also significantly influence the results. Although methodological and reporting issues are common in the field of VR utilization in psychiatry (40), these factors collectively hinder the ability to generalize or draw firm conclusions about the factors influencing offset detection.

Furthermore, while *matching* and *self-presentation* are recognized as crucial factors in immersion and presence, measures of presence or avatar embodiment were employed inconsistently. Only five studies utilized a measure of presence or avatar embodiment, three tested the feeling of presence within the virtual environment, and only three included embodiment questionnaires. A proprioceptive drift self-localization task was performed in just two studies. Both presence and embodiment measures were included in only two studies. This scarcity of data represents a missed opportunity to explore the interrelations between offset detection, immersion, and presence, which are vital aspects of VR interventions.

Lastly, the impact of technological and methodological factors on experimental results should not be overlooked. These factors include, but are not limited to, errors in positional tracking, data losses due to software malfunctions, biases arising from incorrect equipment placement or assembly, and inconsistencies in spatial anchoring of participants with data cables. To illustrate these challenges, in a study by Burns et al. (21), complete data were available for only 19 out of 40 participants, highlighting the significant effect such issues can have on the integrity and interpretability of research findings.

### 4.2 Limitations of the systematic review

The limitations of this systematic review should be acknowledged. The review is confined to reports published in English and was conducted across only two databases, with the search query limited to titles, abstracts, and keywords. Despite the intention to use the most general terms, it is possible that some relevant reports were not identified. Furthermore, during the screening of results, there is a chance that some reports meeting the eligibility criteria may have been inadvertently overlooked. Additionally, the diversity of methods used across the reported studies complicates the task of standardizing the limits for acceptable positional offsets.

### 4.3 Recommendations for experimental design

The insights from studies on offset detection can significantly inform and enhance the experimental designs and reporting practices for VR assessments in mental health research. Utilizing detection thresholds as a practical, technology-agnostic proxy measure allows for the comparison of results across various VR headsets, essential due to the rapid advancements and frequent updates in VR technology. This approach not only addresses the variability introduced by differing technologies but also improves the robustness and applicability of research findings in real-world clinical settings.

In studies exploring positional offsets, body ownership and multisensory integration, it may be recommended to utilize more precise outside-in detection systems than built-in inside-out systems. Calibration of equipment for each individual user, as recommended by Zenner et al. (25) and implemented by Benda et al. (22), is advised to enhance data reliability. Additionally, procedural unification could be further advanced by incorporating standardized questionnaires such as the Virtual Embodiment Questionnaire (VEQ) or the Igroup Presence Questionnaire (IPQ), which provide a foundation for comparable data across different studies.

## 5 Conclusion

Proper alignment between a virtual and a real hand is critical for immersion and presence in virtual reality (VR), which in turn could influence the efficacy of VR mental health interventions. This systematic review reveals a surprisingly wide tolerance for detecting positional offsets within a virtual environment among healthy individuals. While the full extent of this tolerance is not yet completely understood, it appears to be significantly shaped by the design of the application itself, such as the nature of misalignment and the depiction of the virtual hand, and to a lesser extent, by individual factors. Additionally, the accuracy of VR devices plays a crucial role in influencing perception and the requirements for appropriate matching.

The identified influencing factors must be carefully considered to ensure the highest possible user presence in forthcoming therapeutic approaches. Furthermore, the variation in VR device capabilities underscores the importance of reporting device properties, particularly tracking sensitivity, in future research and VR interventions.

Lastly, the existence of individual variability in detecting positional offsets underscores the potential of VR as an optimal tool for researching body ownership and multisensory integration. This has significant implications for developing diagnostic applications, particularly for disorders like schizophrenia, where such research can provide valuable insights.

## Data Availability

The original contributions presented in the study are included in the article/**Supplementary Material**. Further inquiries can be directed to the corresponding author.
